# Yata Virus (Family *Rhabdoviridae*, Genus *Ephemerovirus*) Isolation from Mosquitoes from Uganda, the First Reported Isolation since 1969

**DOI:** 10.3390/diseases11010021

**Published:** 2023-01-28

**Authors:** Lara C. Perinet, John-Paul Mutebi, Ann M. Powers, Julius J. Lutwama, Eric C. Mossel

**Affiliations:** 1 Division of Vector-Borne Diseases, US Centers for Disease Control and Prevention, Fort Collins, CO 80521, USA; 2 Department of Arbovirology, Emerging, and Re-emerging Diseases, Uganda Virus Research Institute, Entebbe, Uganda

**Keywords:** arbovirus, Rhabdoviridae, Ephemerovirus, virus discovery, virus surveillance

## Abstract

As a part of a systematic study of mosquitoes and associated viruses in Uganda, a virus was isolated from a pool of *Mansonia uniformis* collected in July 2017, in the Kitgum District of northern Uganda. Sequence analysis determined that the virus is Yata virus (YATAV; *Ephemerovirus yata*; family *Rhabdoviridae*). The only previous reported isolation of YATAV was in 1969 in Birao, Central African Republic, also from *Ma. uniformis* mosquitoes. The current sequence is over 99% identical at the nucleotide level to the original isolate, indicating a high level of YATAV genomic stability.

## 1. Introduction

*Rhabdoviridae* is a globally distributed, diverse family of viruses comprising 275 species in 45 genera [1]. Rhabdoviruses may be found in both aquatic and terrestrial environments and are known to infect vertebrates, invertebrates, and plants [2]. Vector-borne rhabdoviruses are primarily transmitted by mosquitoes and mites [3]. Due to their potential impact on cattle, fish hatcheries, and human health, several viruses in this family are considered to be of consequence, including rabies and vesicular stomatitis viruses [4]. Rhabdoviruses have a negative-sense RNA genome of 10.8–16.1 kb, usually contained in a single segment [1]. Most viruses in this family contain five conserved structural protein genes: nucleoprotein (N), polymerase-associated phosphoprotein (P), matrix protein (M), glycoprotein (G) and RNA-dependent RNA polymerase (L) [3]. Many also contain a variety of accessory genes encoded between the canonical genes and/or in alternate overlapping open reading frames, contributing to the diversity of the family [2,5].

The *Ephemerovirus* genus includes some of the largest viruses in the *Rhabdoviridae* family due to their relatively complex genomes [3]. In addition to the five canonical structural proteins, ephemeroviruses may have up to six accessory genes, found in between the G and L genes [5]. This genus currently includes 11 species, each comprised of a single virus, which have been identified in tropical and subtropical regions in Africa, Asia, and Australia [2,6]. These are typically found in and around livestock and are primarily transmitted by arthropods, specifically mosquitoes and biting midges [5]. Bovine ephemeral fever virus (BEFV), for which the *Ephemerovirus* genus is named, is the most well-characterized of the ephemeroviruses. It is found extensively in Africa, Asia and Australia, and it causes severe, debilitating, febrile illness in cattle and water buffalo [7]. Outbreaks occur seasonally and can have a large impact on the dairy industry [7,8]. Due to their potential impact on livestock and widespread distribution, it is important to continue to improve our understanding of viruses in this genus.

**Figure 1:**
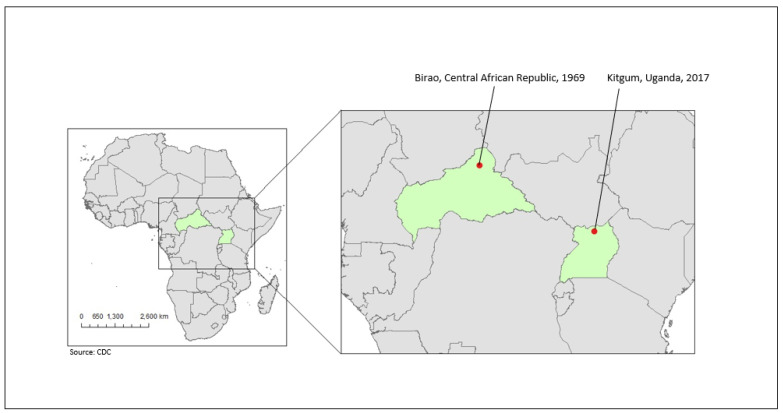
Location and year of collection of *Mansonia uniformis* mosquitoes yielding Yata virus isolates.

The only reported isolation of Yata virus (*Ephemerovirus yata*; YATAV) occurred in 1969 from a pool of *Mansonia uniformis* mosquitoes collected near Birao, Central African Republic (Figure 1) [9]. Though initially suspected to be a rhabdovirus, YATAV failed to react in classical serological assays with other rhabdoviruses, making its classification unclear [10,11]. In 2014, the complete YATAV genome was determined using next generation sequencing (NGS) technology, which confirmed its status as a rhabdovirus and further resulted in its classification as an ephemerovirus [12]. With a total of 14,778 nucleotides, the YATAV genome is the smallest among the known ephemeroviruses. The Yata virus genome follows a typical ephemerovirus structure, with five structural protein genes and six accessory genes located between the G and L genes. Of the six accessory genes, only two have known functions [12]. Notably, the G_NS_ accessory gene encodes a class 1 transmembrane glycoprotein, which is largely homologous with other ephemerovirus G_NS_ proteins. The α1 gene encodes a transmembrane protein, which has the structural characteristics of class I viroporins [12]. Little else is known about YATAV. No animal host, reservoir, or geographic range has been determined, nor have any other isolates been reported. Here, we describe the isolation and molecular characterization of a second YATAV isolate, obtained from *Ma. uniformis* mosquitoes collected in Uganda in 2017.

## 2. Materials and Methods

### 2.1. Mosquito Trituration and Infection Status Determination

Mosquitoes were identified, pooled, and processed for the isolation of viruses as detailed in Mossel et al. [13]. Briefly, pools of ≤25 mosquitoes were separated by date and location of collection, species, gender, and gravid/engorged status, and were triturated. Clarified homogenate was used to inoculate Vero cell monolayers in 6-well tissue culture plates. Plates were monitored for plaque formation for as long as monolayer viability allowed or up to two weeks. Supernatants within wells in which plaques were observed were harvested, and collected material was used to inoculate T-25 flasks of Vero cells to amplify virus. Flasks were monitored daily, and the supernatants were harvested when the monolayers showed a clear cytopathic effect.

### 2.2. RNA Sequencing

RNA was extracted from cell culture supernatant (QIAamp Viral RNA Mini Kit, Qiagen, Valencia, CA, USA). DNase-treated RNA was reverse-transcribed (New England Biolabs OneTaq RT-PCR Kit, Ipswich, MA, USA) into cDNA, amplified, and sequenced using a MinION Mk1C and Ligation Sequencing Kit (Oxford Nanopore Technologies, London, UK; ONT). Reads were identified using integrated ONT software, Epi2Me, and confirmed by NCBI BLAST search.

Using the YATAV sequence of Blasdell et al. [12] (GenBank accession number NC_028241.1), PCR primers were designed to generate amplicons of approximately 2 kb having approximately 200 bp overlap and covering the complete coding region. After clean-up, amplicons were combined for multiplex sequencing. Library prep and sequencing was performed using a MinION Mk1C and Ligation Sequencing Kit (ONT). The process was repeated to obtain a sufficient depth of coverage across the genome.

A de novo and referenced assembly of reads into contigs was performed using CLC Genomics Workbench ver. 20 (Qiagen). The phylogenetic relationship of YATAV UG12209 to other ephemeroviruses was determined by a maximum likelihood analysis of the nucleic acid sequence using ClustalW alignment and the Tamura–Nei model with 1000 iterations on MEGAX software [14].

## 3. Results

As part of an arbovirus surveillance project in East Africa, mosquitoes were collected from various ecological niches during both the rainy and dry seasons in Uganda to be tested for the presence of replication-competent virus by a plaque assay on Vero cells [13]. The homogenate of a pool of 25 female *Ma. uniformis* collected using CDC light traps in July 2017 from Ogwapoke village, Kitgum District in north-central Uganda (Figure 1) generated plaques 10 days after cell-culture inoculation. The preliminary NGS analysis of the extracted RNA resulted in a presumptive YATAV identification. For confirmation, PCR was performed using two primer sets developed from the 2014 sequence of Blasdell et al. (GenBank accession number NC_028241) [12], each directed toward the 5′ and 3′ ends of the genome. The resulting amplicons were of the expected size and confirmed the identity of the isolate as YATAV (not shown). To fill in sequence gaps and increase the depth of coverage to the preliminary sequence, additional PCR primers were designed across the YATV genome based on our preliminary sequence and the published sequence. Amplicon sequencing on the ONT Mk1C resulted in a total of over 3 million reads for the eight PCR amplicons with a depth of coverage of up to 40,000 reads. A referenced sequence assembly of the resulting reads, trimmed to remove primer sequences, was performed using CLC Genomics Workbench version 20 (Qiagen).

**Figure 2:**
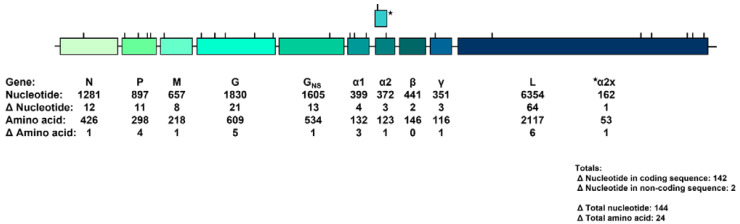
Yata virus genome with nucleotide and amino acid difference between the YATAV isolate UG12209 and the original isolate, DakArB 2181. Tick marks represent the relative locations of amino acid differences in each open reading frame.

**Figure 3:**
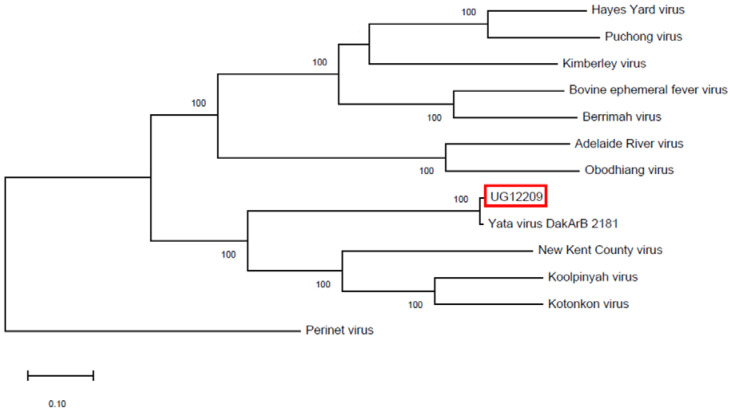
Nucleotide maximum-likelihood phylogenetic tree with 1000 bootstrap replications comparing the two reported Yata virus isolates to other known ephemeroviruses and rooted with Perinet virus (genus *Vesiculovirus*). The current isolate, 12209, is boxed in red. GenBank accession numbers: Hayes Yard virus—MH507506.1, Puchong virus—MH507505.1, Kimberley virus—NC_025396.1, Bovine ephemeral fever virus—MW512963.1, Berrimah virus—NC_025358.1, Adelaide River virus—NC_028246.1, Obodhiang virus—NC_017685.1, Yata virus (UG12209)—ON375848.1, Yata virus (DakArB 2181)—NC_028241.1, New Kent County virus—MF615270.1, Koolpinyah virus—NC_028239.1, Kotonkon virus—DQ457099, Perinet virus—NC 025394.1.

Following assembly, a BLAST analysis of the 14,478-nucleotides-near-full-length sequence returned a 99.01% nucleotide identity with YATAV DakArB 2181. Sequence alignment revealed 144 nucleotide differences across the genome, with 142 in the coding sequence. The two isolates differed by seventeen amino acids in the five canonical structural genes and by an additional six in the accessory genes, indicating that most nucleotide changes were silent. No pattern or clustering of differences was obvious, with differences appearing to be randomly dispersed throughout the genome (Figure 2). One or more missense amino acid mutations were found in all genes, including the accessory genes, except the β accessory gene. A nucleotide sequence maximum-likelihood analysis confirmed the relationship of the YATAV isolates to each other and within the *Ephemerovirus* genus (Figure 3).

## 4. Discussion

We report the isolation of a little studied ephemerovirus, YATAV. As only the second known isolation of this virus, 48 years removed from the original, this isolation offers an opportunity to examine the change over time of the YATAV genome. Rhabdoviruses are known to mutate quickly due to an error-prone RNA-dependent RNA polymerase and adaptation resulting from selective pressures [15,16]. While the rate of evolution of most ephemeroviruses remains unclear [6], BEFV was found to have an evolutionary rate of approximately 10^−3^ substitutions/nucleotide/year (s/n/y) [17]. The current YATAV isolate differs by less than 1% at the nucleotide level from the original isolate, despite the temporal and geographic distance between the two, a suggested mutation rate of approximately 2 × 10^−4^ s/n/y. The majority of the changes were silent, resulting in only 24 amino acid differences. Such similarity suggests that the virus likely persists in a relatively stable transmission cycle. Additional sequences, particularly those that may be obtained from other vectors and/or hosts, will help refine the YATAV rate of evolution. Further study of the effect of the nucleotide and amino acid differences is also needed to understand what, if any, functional and antigenic effects these changes might have on the viral life cycle.

Phylogenetic analysis demonstrates that ephemeroviruses can be separated into two clades based on the nucleotide sequence (Figure 3). One clade includes bovine ephemeral fever virus and related viruses. Yata virus belongs to the diverse second clade, along with New Kent County virus, Koolpinyah virus, and Kotonkan virus (KOTV). New Kent County virus was detected in *Ixodes scapularis* ticks in the United States and is known only by sequence. Koolpinyah virus was isolated from cattle in Australia, and an arthropod vector has not yet been identified [18]. Kotonkan virus was isolated from *Culicoides* spp. in Nigeria but may be primarily vectored by mosquitoes [12]. Serosurveys detected KOTV-neutralizing antibody in humans, cattle, sheep, horses, and rodents [19]. Kotonkan virus was also associated with potentially fatal disease in naturally and experimentally infected dairy cattle [19,20]. Yata virus has now been isolated twice from *Ma. uniformis* mosquitoes but has yet to be associated with the infection of vertebrates. The virus was isolated and amplified in Vero cells, indicating that it is not an insect-only virus. Further, based on blood meal analyses and bait preferences, *Ma. uniformis* is known to feed on vertebrates, particularly mammals, possibly preferring humans [21,22,23,24,25,26]. Thus, while the importance and status of YATAV as an arbovirus is not certain [27], evidence from its nearest neighbors and from what is known of its potential vector supports this possibility. Serosurveys in Central-East Africa in humans, livestock, and wild animals will be necessary to determine the host range and pathological importance of YATAV.

The results presented herein are part of a larger study with the aim of understanding the circulation of arboviruses throughout Uganda by collecting mosquitoes in various ecological niches at different times of the year over multiple years and analyzing them for the presence of replication-competent viruses. The past decade has seen numerous large viral epidemics and pandemics, several of which have been caused by arboviruses: Zika virus, chikungunya virus, and yellow fever virus, to name a few. The results of surveillance studies such as this will increase our understanding of the viral emergence potential and possibly suggest lines of effort toward diagnostic development and control measures for these viruses.

## Data Availability

The data presented in this study are available on request from the corresponding author.
